# Network Anomaly Detection System with Optimized DS Evidence Theory

**DOI:** 10.1155/2014/753659

**Published:** 2014-08-31

**Authors:** Yuan Liu, Xiaofeng Wang, Kaiyu Liu

**Affiliations:** ^1^School of Digital Media, Jiangnan University, Wuxi, Jiangsu 214122, China; ^2^School of Internet of Things Engineering, Jiangnan University, Wuxi, Jiangsu 214122, China

## Abstract

Network anomaly detection has been focused on by more people with the fast development of computer network. Some researchers utilized fusion method and DS evidence theory to do network anomaly detection but with low performance, and they did not consider features of network—complicated and varied. To achieve high detection rate, we present a novel network anomaly detection system with optimized Dempster-Shafer evidence theory (ODS) and regression basic probability assignment (RBPA) function. In this model, we add weights for each senor to optimize DS evidence theory according to its previous predict accuracy. And RBPA employs sensor's regression ability to address complex network. By four kinds of experiments, we find that our novel network anomaly detection model has a better detection rate, and RBPA as well as ODS optimization methods can improve system performance significantly.

## 1. Introduction

With the development of computer network technology and the increasing of the networks scale, computer networks are under the threat of attack from hackers and other technologies, so the security status of the computer networks is becoming the focus of people's attention. Intrusion detection technology, protecting the network security behind the firewall, is becoming the research focus in the recent network security field. As the emphasis and difficulty of the network intrusion detection technology [1], network anomaly detection technology has the deficiency of the low detection rate, high false positive rate, and high false negative rate at present. So in this domain, many researchers proposed lots of useful algorithms [2–8], but these methods are so simple and single that they cannot be fully adapted to complicated and changeable network. Thus, a novel network anomaly detection mechanism is required to solve the above troubling problems.

Recently, some researches cope with network anomaly detection utilized by Dempster-Shafer (DS) evidence theory [9, 10] proposed by Dempster in 1976 and then improved by his student Shafer, which has been widely used in many fields of data fusion, such as expert advisory system, forecasting, image processing, artificial intelligence, and identifying classification. Intrusion detection is a problem of multiclassification essentially, which divides network data into normal data and various types of attacking data. Since simple detection algorithms always suffer from limitations such as low detection rate and high false alarm rate, many researchers apply DS evidence theory into intrusion detection systems. For example, some researchers divide the characteristics of network data into the basic feature set, the content feature set and the traffic feature set. Then they utilize detection algorithm to detect these three feature sets and fuse data through DS evident theory to get the final results. Though the IDS theory based on DS evidence theory has a good detection rate, most of these studies based on the classic DS evidence theory should assume that the intercepted data is independent of each other without confliction. However, conflicts between network data are inevitable, so those researches will lead to unreasonable fusion result, high false alarm rate and miss alarm rate.

To better solve this serious issue, we present a novel network anomaly detection mechanism based on optimized DS evidence theory (ODS) which also can achieve better reasonable result in network conflict data, unlike conventional DS evidence theory. In this mechanism, we employ ODS to merge 3 classifiers, support vector machine classifier (SVM) [11, 12], biased minimax probability machine classifier (BMPM) [13], and back propagation network classifier (BP) [14]. Unlike the original fusion rule using the classification feature of those classifiers, the new one utilizes its regression feature, because regression feature can better reflect real-time network environment. Note that, since network environment is almost complicated and varied, in DS evidence theory, each sensor cannot be equally computed. So we assign different weights for each sensor, respectively, according to its previous prediction accuracy. In addition, based on different distance sizes between network connections, we present a new construction method for basic probability assignment function (BPA) based on regression ability, regression BPA (RBPA), unlike simple BPA (SBPA). Finally, through comparison of 4 fusion methods and 3 single methods, the experimental results with KDD99 [15] show that the ODS algorithm can overcome the conflict problem among the evidences, and the proposed module can improve the detection performance of the anomaly detection system.

The reminder of this paper is organized as follows. In Section 2, we will introduce class DS evidence theory, analyze its limitation, and present an optimized DS evidence theory with weights for each sensor. Then we propose a novel network anomaly detection model with ODS in Section 3. In Section 4, we introduce 4 key issues: how to combine ODS evidence theory with network anomaly detection, how to construct and decide BPA value in ODS evidence theory, how to decide weight *w*_*i*_ in fusion rules of ODS evidence theory, and how to train 6 classifiers. With data set KDD99, we evaluate our novel network anomaly detection model by 4 kinds of experiments in Section 5. Next, Section 6 introduces related work. Finally, we conclude our main work and propose future work in Section 7.

## 2. Optimized DS Evidence Theory

### 2.1. Class DS Evidence Theory

DS evidence theory [9, 10] is considered as a general extension of the traditional classical probabilistic inference theory in the finite field. Unlike the conventional Bayes inference method, DS evidence theory without a priori probability still can be used to deal with uncertainty and imprecision information. So we can see that DS evidence theory has greater flexibility.

DS evidence theory is considered as theory built on a nonempty finite field Θ called the recognition framework, which includes a limited number of independent system state {*A*_1_, *A*_2_, …, *A*_*n*_}. An element in *P*(Θ) as a power set of system state Θ is called a system state hypothesis *H*_*i*_. Through the observation results *E*_1_, *E*_2_,…, *E*_*m*_ for system state by each sensor, DS evidence theory can merge these results and infer the former state of system. Here it mainly involves the following concepts.

**Definition 1 .**

Basic probability assignment function (BPA) is defined as a map from a power set of Θ to [0,1] interval. It is represented as *m* : *P*(Θ)→[0,1], *m*(Φ) = 0, ∑_*A*∈*P*(Θ)_ *m*(*A*) = 1, where *m*(*A*) is called confidence value which means that current sensor decides hypothesis *A* the degree of confidence according to the observation results.

**Definition 2 .**

Belief function is defined as

(1) $$\begin{array}{c} \text{Belie}{\text{f}}_{i}\left(A\right)=\underset{E_{k}\subseteq A}{\sum }m_{i}\left(E_{k}\right). \end{array}$$

This function represents the degree of confidence for hypothesis *A*. And the function result is composed of basic confidence values of observation results *E*_*k*_ which supports hypothesis *A*.

**Definition 3 .**

Plausibility function is defined as

(2) $$\begin{array}{c} \text{Plausibilit}{\text{y}}_{i}\left(A\right)=1-\underset{E_{k}\cap A=\Phi }{\sum }m_{i}\left(E_{k}\right). \end{array}$$

This function represents the degree of plausibility for hypothesis *A*. And the function result is composed of basic confidence values of observation results *E*_*k*_ which supports hypothesis *A*.

**Definition 4 .**

DS fusion rules, for any hypothesis *A*, defining *m*_*i*_ and *m*_*j*_ as the basic probability assignment function (BPA) of two evidences, respectively, state that one obtains basic belief assignment function of the combination evidence from two evidences above as follows:

(3) $$\begin{array}{c} \left(m_{i}⊕m_{j}\right)\left(A\right)=\frac{\sum_{E_{k}\cap E_{k^{\prime }}=A}^{}m_{i}\left(E_{k}\right)m_{j}\left(E_{k^{\prime }}\right)}{1-\sum_{E_{k}\cap E_{k^{\prime }}=\emptyset }^{}m_{i}\left(E_{k}\right)m_{j}\left(E_{k^{\prime }}\right)}. \end{array}$$

Likewise, one can achieve DS general synthesis rules for the combination evidence from *n* evidences as follows:

(4) $$\begin{array}{c} m_{1\cdots n}\left(A\right)=\frac{\sum_{\cap_{i}E_{i}=A}^{}m_{1}\left(E_{1}\right)m_{2}\left(E_{2}\right)\cdots m_{n}\left(E_{n}\right)}{\sum_{\cap_{i}E_{i}\neq A}^{}m_{1}\left(E_{1}\right)m_{2}\left(E_{2}\right)\cdots m_{n}\left(E_{n}\right)}. \end{array}$$

### 2.2. Drawback of Class DS Evidence Theory

The advantage of DS evidence theory mainly focuses on several parts as follows: it can satisfy axiom system that is weaker than the probability, distinguish unknown and uncertainty situation, and continuously shrink the hypothesis set in the light of the accumulation of evidences.

The disadvantage of DS evidence theory is that, when dealing with the issue with confidence degree tending to 0, the result computed by DS evidence theory will conflict with expectation result. That is to say, when confidence degree is too small or 0, the results achieved are very different. In the same way, BPA is required to give so many results that calculation is also more complicated. If the hypothesis set is too large, the calculation complexity of evidence theory will increase exponentially.

### 2.3. Optimized DS Evidence Theory

From formula (4), we can see that the confidence degree of each sensor is the same. That is, each sensor has the same accuracy. Obviously, it does not fit the facts, for example, with doctor for treatment. A doctor considers that this patient may be suffering from X disease with 99%, or Y disease with 1%. However, B doctor considers that this patient may be suffering from Y disease with 1%, or Z disease with 99%. Then we can achieve this patient suffering from Y disease according to formula (4) merging these different two pieces evidence. But this result is fully fault and does not match reality. Therefore, the synthesis rule from formula (4) only applies to the case with the same precision in all sensors.

To solve this serious issue, we present a novel method to change with conventional DS evidence theory. That is, we combine weights with DS evidence theory. The detailed implementation is that we add weight value to each sensor according to its previous predict accuracy. So we define *m*_*i*_ as the basic confidence values obtained by sensor *S*_*i*_, and *w*_*i*_ as the previous predict accuracy of sensor *S*_*i*_; similarly, *m*_*j*_ as the basic confidence values obtained by sensor *S*_*j*_, and *w*_*j*_ as the previous predict accuracy of sensor *S*_*j*_. The DS evidence theory combination with weights as follows:

(5) (mi⊕mj)(A) =∑Ek∩Ek′=A[wimi(Ek)·wjmj(Ek′)]1−∑Ek∩Ek′=∅[wimi(Ek)·wjmj(Ek′)].

## 3. ODS Network Anomaly Detection Model Design

**Figure 1:**
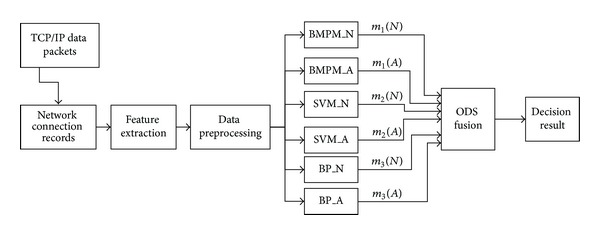
A novel network anomaly detection model with ODS evidence theory.

In this paper, we present a novel network anomaly detection module based on optimized DS evidence theory merging with several kinds of classifiers. In this module, we utilize BMPM, SVM, and BP network as classifiers. Unlike the original fusion rule using the classification feature of those classifiers, the new one utilizes its regression feature, because regression feature can better reflect real-time network environment. Then we consider the merged result as one parameter used to construct BPA of DS evidence theory. And then we will introduce this novel network anomaly detection model in detail, which is depicted in Figure 1.

As shown in Figure 1, this module mainly consists of five modules: network connection record module, feature extraction module, data preprocessing module, early detection module, and ODS fusion module, respectively.

Network connection record module utilizes some network sniffer tools, for example, Sniffer, to collect network packets in the network where network anomaly detection host is, and then stores it. That is, this module is used to collect network data.

Feature extraction module is used to extract some features impacting network anomaly detection, which are in the network packets stored by network connection record module. And then we record corresponding features into a feature vector, in order to preprocessing module to use it. Similarly, this module gets rid of unconcerned features for network anomaly detection. Essentially, this module is used to complete feature reduction.

Data preprocessing module is used to cope with feature vector after feature extraction. In addition, some futures in one feature vector are discrete type, such as protocol type, service type and logo, and others are continuous type, such as connection time type, the length of data sent, and the length of data received. Since discrete data needs inputting into detection module in early phase, in order to following work, continuous features need to be discretized. At the same time, these feature vectors also need to be standardized and normalized, in order for these vectors to be normally operated in BP network. In essence, this module is used to do data normalization, discretization, and standardization.

Early detection module is employed to detect the feature vectors that have processed by data preprocessing module and gives the corresponding detection results for DS fusion module later. It is composed of 3 sensors: SVM, BMPM, and BP. To fit with complicated network environment, we optimize sensors, that is, add weights into each sensor and construct 6 classifiers: BMPM_N, BMPM_A, SVM_N, SVM_A, BP_N, and BP_A (Section 4.3). Then we train these classifiers according to distance theory [16] (Section 4.4). Finally, we achieve several results when a network record is coming.

ODS fusion module will utilize ODS evidence theory to merge and analyze these detection results from early detection module. That is, according to regression ability of sensors, we fuse these results by (15) and give decision results, that is, whether the attack or not.

## 4. ODS Network Anomaly Detection Model Implementation

In ODS network anomaly detection model, we should solve several key issues: how to combine ODS evidence theory with network anomaly detection, how to construct and decide BPA value in ODS evidence theory, how to decide weight *w*_*i*_ in fusion rules of ODS evidence theory, and how to train 6 classifiers in detail, and so forth. Therefore, in this section, we will introduce the solutions for these serous issues mentioned above in detail.

### 4.1. Combining ODS Evidence Theory with Network Anomaly Detection

Since in ODS network anomaly detection model, system judges that whether current connection is unusual only according to network feature observed, we only define ODS evidence theory identification framework with two elements: normal status and abnormal status.

Therefore, according to DS evidence theory, we define ODS evidence theory identification framework as {*N*, *A*}, where *N* represents normal status and *A* represents abnormal status. We can see that status *N* and *A* are mutual exclusion, that is, *N*∩*A* = *∅*. Similarly, we can redefine BPA function as *m* : *P*({*N*, *A*})→[0,1], *m*(*∅*) = 0, *m*({*N*, *A*}) + *m*(*N*) + *m*(*A*) = 1. In above formula, *m*(*N*) represents the observation results of current feature by current sensor and considers that reliability of current status belongs to abnormal status. On the other hand, *m*({*N*, *A*}) represents the observation results of current feature by current sensor and cannot decide reliability of current status belongs to normal or abnormal status. We will introduce detailed BPA function in next subsection.

### 4.2. Regression BPA

In this subsection, we first give a hypothesis about network connection status for BPA value and then depict sensors' regression ability and how to compute RBPA value in detail.

#### 4.2.1. Hypothesis and Discussion for Network Connection Status

Reference [16] said that the distance between abnormal network connection and normal network connection is larger than that between normal network connection and normal network connection. That is, for classifiers, the distance of different data is larger than that of same data. According to this rule, here we give a hypothesis: for a network connection to be seen (unknown), the prediction result will be *m*(*N*) with N-classifier. That is to say, N-classifier considers all the network connection as normal network connection all the time, but only gives corresponding different support degrees according to difference of real network connection: high support degree for real normal network connection and low support degree for real abnormal connection. Through this hypothesis, we can see that for a real normal network connection, the prediction result *m*(*N*) computed by N-classifier is larger than *m*(*A*) computed by A-classifier and vice versa.

From Figure 1, three kinds of classifiers in early detection module, such as SVM, BMPM, and BP are also considered as three sensors. SVM_N and SVM_A are, respectively, represented support degree of normal and abnormal network connection from SVM sensor. Similarly, this rule is also suitable for BMPM and BP. So when we assign the same parameter for SVM_N and SVM_A, respectively, they can be considered as a whole sensor. And this whole sensor can give different support degrees to normal and abnormal network connection, respectively. Similarly, this is suitable for BMPM and BP sensor. Therefore in this novel model, the fusion part is considered as ODS evidence theory combining with three kinds of sensors, SVM, BMPM, and BP. If a normal network connection needs processing, no matter which senor (SVM, BMPM, and BP), the support degrees *m*(*N*) and *m*(*A*) for this network connection are, respectively, achieved by the chosen sensor. And these results satisfy the objective fact of this network connection. That is, in ODS evidence theory, if  *m*(*N*) is larger than *m*(*A*), this network connection is considered as a normal one.

Based on this hypothesis above, if this novel model is required to automatically give current network connection support degree of normal and abnormal status by each sensor, and this result can also satisfy the objective fact of real network connection, we will utilize the features of sensors, such as study ability, regression ability, associative memory ability, and generalization ability. That is because sensors (e.g., BMPM, SVM, and BP) can achieve similar result after training, learning, and regression operations, which is almost equal to the actual result. So we see that sensors with their features can reflect the real network environment.

#### 4.2.2. The Features of Sensors

In the above subsection, we said that some features of sensors will be selected to help with BPA function construction, so here we utilize regression ability, supervised learning ability of SVM, BMPM, and BP sensor. That can be explained in detail as follows: here we define one class of data as normal network connection data *N* and its corresponding training data *NT*, and define another class of data as abnormal network connection data *A* and its corresponding training data *AT*. Then these two kinds of data and their corresponding training data are used to train these classifiers depicted in Figure 1. As long as the two kinds of data distribution and training data have obvious difference, when a data record satisfies any kinds of data mentioned above, we can estimate the value of this data record (*NT* or *AT* corresponding with training data) utilizing the regression ability of these classifiers. In addition, the estimate values show obvious difference due to data record satisfying different kinds of data distribution.

#### 4.2.3. BPA Based on Regression Ability

Since network connection status can be represented as normal or abnormal status by different sensors which give different support degrees for them, with this rule, we construct BPA function in ODS network anomaly detection model. When ODS evidence theory is combined with network anomaly detection, assuming that the current network connection is a normal one, corresponding BPA value can be different achieved by different sensors (various classifiers in fusion model). And the BPA values are corresponding with hypothesis *N*, *A* or {*N*, *A*}. Similarly, we also expect that the BPA value of normal network connection assigned by hypothesis *N* is larger, but on the contrary, the BPA value assigned by hypothesis *A* (abnormal status) or hypothesis {*N*, *A*} (unknown status) should be smaller.

After training N-classifier and A-classifier (training classifiers will be introduced in Section 4.4), we can compute BPA value in ODS evidence theory. Currently, SVM_N and SVM_A can be considered as a whole one, a SVM sensor. For a network connection record, the regression estimates value *m*(*N*) computed by SVM_N and *m*(*A*) computed by SVM_A. Due to associated ability of SVM classifier, if this record is a normal network connection record, *m*(*N*) will be larger than *m*(*A*), vice versa. In addition, this rule is also suitable for BMPM and BP. Therefore, these three sensors, SVM, BMPM, and BP, in system can assess current status for a coming network connection. That is, we can achieve support degrees *m*(*N*) and *m*(*A*) for normal status and abnormal status, respectively.

Noticeably, in this paper we present a novel method to deal with unknown network connection status as follows:

(6) $$\begin{array}{c} m\left(\left\{N,A\right\}\right)=\{\begin{array}{cc} 1-m\left(N\right)-m\left(A\right) & \;m\left(N\right)+m\left(A\right)<1\\ 0 & m\left(N\right)+m\left(A\right)\geq 1. \end{array} \end{array}$$

From formula (6), we can see that when *m*(*N*) + *m*(*A*) < 1, we define *m*({*N*, *A*})*m*({*N*, *A*}). Similarly, when *m*(*N*) + *m*(*A*) ≥ 1, the value of *m*({*N*, *A*}) is 0. *m*(*N*) and *m*(*A*) should be normalized at the same time. Thus, this is suitable for the requirement of ODS evidence theory *m*(*N*) + *m*(*A*) + *m*({*N*, *A*}) = 1, and the computation of RBPA function is completed in ODS evidence theory.

### 4.3. Weights for Each Sensor

In the traditional network anomaly detection system, the performance is decided by an estimate parameter* F-Score*, which reflects that an intrusion detection system performance is good or bad. And the greater the* F-Score* indicates that the better performance of this system. So in this paper, we extend this important parameter* F-Score* and propose two new parameters,* F-Score-N* and* F-Score-A*. Since with ODS evidence theory, the *w*_*i*_ value depends on its previous accuracy in the process of sensor prediction, we utilize these new parameters to add weights for each senor. Then we will introduce these new parameters in detail.

Here, we define several parameters, respectively, as follows:

- TP: the number of abnormal connection detected by anomaly detection system (abnormal connection itself);
- FN: the number of normal connection detected by anomaly detection system (abnormal connection itself);
- FP: the number of abnormal connection detected by anomaly detection system (normal connection itself);
- TN: the number of normal connection detected by anomaly detection system (normal connection itself);
- Precision: the proportion of true abnormal connections of abnormal connections detected by anomaly detection system;
- Recall: the proportion of abnormal connections detected by anomaly detection system of true abnormal connections;
- *F-Score*: a balance average parameter for Precision and Recall used to estimate a network anomaly detection system.

With these parameters mentioned above, the formulas are depicted as follows:

(7) $$\begin{array}{c} \text{Precision}=\frac{\text{TP}}{\text{TP}+\text{FP}},\\ \text{Recall}=\frac{\text{TP}}{\text{TP}+\text{FN}},\\ F\text{-}Score=\frac{2*\text{Precision}*\text{Recall}}{\text{Precision}+\text{Recall}}. \end{array}$$

But these conventional formulas do not satisfy this novel network anomaly detection system presented in this paper, so we propose these new parameters as follows:

- Precision_A: The proportion of abnormal connections of abnormal connections detected by anomaly detection system (abnormal connection itself);
- Recall_A: The proportion of abnormal connections detected by anomaly detection system of all true abnormal connections (abnormal connection itself);
- Precision_N: The proportion of normal connections of abnormal connections detected by anomaly detection system (normal connection itself);
- Recall_N: The proportion of normal connections detected by anomaly detection system of all true normal connections (normal connection itself);
- *F-Score-N*: The accuracy for normal connections estimated by sensors, that is, N-classifiers' weights;
- *F-Score-A*: The accuracy for abnormal connections estimated by sensors, that is, A-classifiers' weights.

With these parameters mentioned above, the formulas are depicted as follows:

(8) $$\begin{array}{c} \text{Precision}\_\text{A}=\frac{\text{TP}}{\text{TP}+\text{FP}}, \end{array}$$

(9) $$\begin{array}{c} \text{Precision}\_\text{N}=\frac{\text{TN}}{\text{TN}+\text{FN}}, \end{array}$$

(10) $$\begin{array}{c} \text{Recall}\_\text{A}=\frac{\text{TP}}{\text{TP}+\text{FN}}, \end{array}$$

(11) $$\begin{array}{c} \text{Recall}\_\text{N}=\frac{\text{TN}}{\text{TN}+\text{FP}}, \end{array}$$

(12) $$\begin{array}{c} F\text{-}Score\text{-}N=\frac{2*\text{Precision}\_\text{N}*\text{Recall}\_\text{N}}{\text{Precision}\_\text{N}+\text{Recall}\_\text{N}}, \end{array}$$

(13) $$\begin{array}{c} F\text{-}Score\text{-}A=\frac{2*\text{Precision}\_\text{A}*\text{Recall}\_\text{A}}{\text{Precision}\_\text{A}+\text{Recall}\_\text{A}}. \end{array}$$

### 4.4. Training Classifiers

In this subsection, we mainly introduce how to train 6 different classifiers including collecting training data set, preprocessing training data set, and training classifiers.

#### 4.4.1. Collecting Training Data Set

First, collecting training data set is introduced in detail. Here we simulate network attack to attack this system and implement network connection record module at the same time. And then these records are stored. The detailed process is depicted as follows.

1. We utilize corresponding attack software to simulate all kinds of attacks, for example, DOS attack which can be simulated by the combination DOS attack simulator with ping command, and others also can be achieved by this way.
2. Before these abnormal connections attacking system, we should record IP address and attack types of attack hosts, respectively, IP address of destination hosts, and simultaneously implement network connection record module in network anomaly detection system to record network packets. Here the time window for attack time is 1 hour, and network connection record module also records all the network packets in this period.
3. Based on these network packets recorded, we filter these network packets according to effective network attack packet standard corresponding with IP address and attack type of attack host recorded before attacking. Then these filtered network packets should be discretized, standardized, and normalized by feature extraction module and data preprocessing module, respectively, in Figure 1. Finally, we can obtain different kinds of network connection feature vectors, such as Normal, Dos, Probe, R2L, and U2R.

#### 4.4.2. Preprocessing Training Data Set

According to the rule mentioned in [16], we utilize this different distance for same or different kinds of network connection to define BPA value in ODS evidence theory. Before train 6 classifiers, we must preprocess this training data set.

As shown in Figure 1, we can see that six basic classifiers in early detection module can be divided into two categories, namely, N-classifier and A-classifier. Before training N-classifier and A-classifier, we should preprocess training data set, and this will be introduced in detail. First, we analyze training N-classifier as follows.

1. When a training data set includes normal connections, N-Train and abnormal connections, A-Train, it should be processed before training N-classifier. First, we compute clustering center, N-CORE of N-Train in training data set.
2. Then we should compute the distance between this normal connection and N-CORE and define this distance value as a positive value, which also corresponds with this normal connection.
3. Then we should compute the distance between this abnormal connection and N-CORE and define this distance value as a negative value, which also corresponds with this abnormal connection.
4. Finally, the results from (2) and (3) are stored into N-Dist which corresponds with connection records. And this list N-Dist will be normalized from 0 to 1 and is considered as a training label to train N-classifier.

After processing above, distance corresponding with normal connection is larger than that with abnormal connection in training data set. If these distance values are used as supervised learning training label when training classifiers, these classifiers will learn this phenomenon through associated ability. So we can see that the regression value for normal network connection will be larger than that for abnormal network connection, when a normal network connection and an abnormal network connection need processing. Indeed, this rule mentioned above is also suitable for training A-classifier.

Then we will train 6 classifiers: N-classifiers utilizing training data set and corresponding N-Dist, A-classifiers utilizing training data set and corresponding A-Dist.

#### 4.4.3. Training Classifiers

In this phase, we mainly train 6 classifiers in early detection module and compute some parameters in ODS fusion module. Here we divide network connection feature vectors (Normal, Dos, Probe, R2L, and U2R) into two parts according to attack type. Each part includes processed training data set and corresponding list (N-Dist, A-Dist) stored distance value.

1. One part is used to train 6 classifiers, SVM_N, SVM_A, BMPM_N, BMPM_A, BP_N, and BP_A, and then these classifiers trained will be stored to do prediction in future.
2. Another part is employed to predict these trained classifiers, and it should record these results including all kinds of attacks and normal connections, that is, these results for TP, TN, FP, and FN depicted in Section 4.3.
3. Then we can get weights* F-Score-N* and* F-Score-A* of all sensors, SVM, BMPM, and BP computed by formulas (12) and (13). Finally, these values are stored into array* F-Score-N* and* F-Score-A,* respectively.

### 4.5. Execution Flow of ODS Network Anomaly Detection System

After training 6 classifiers introduced in Section 4.4, we can easily get weights for each sensor (in Section 4.3). As the same, the BPA values of each sensor, support degree *m*(*N*), *m*(*A*), and *m*({*N*, *A*}), can be achieved easily introduced in Section 4.2. In this section, execution flow of ODS network anomaly detection system will be introduced in detail.

(1) First, we can get a network connection packet by network connection record module, and then a network connection feature vector can be obtained by feature extraction module and data preprocessing module which process the network connection packet achieved one by one.
(2) Then this network connection feature vector will be processed to do regression estimate by 6 classifiers (3 sensors) in early detection module. So we can obtain support degree *m*_1_(*N*) and *m*_1_(*A*) for normal network connection status and abnormal network connection status after sensor SVM processing this network connection. Next computed by formula (6), support degree *m*_1_({*N*, *A*}) for unknown status is also achieved easily.
(3) In this way, we can be easy to obtain *m*_2_(*N*), *m*_2_(*A*), and *m*_2_({*N*, *A*}) corresponding with BMPM, and *m*_3_(*N*), *m*_3_(*A*), and *m*_3_({*N*, *A*}) corresponding with BP.
(4) Here we achieve an ODS evidence theory with weights for *n*-sensors inferred by formulas (4) and (5):
  (14) m1⋯n(A) =∑∩iEi=Aw1·m1(E1)·w2·m2·(E2)⋯·wn·mn(En)∑∩iEi≠Aw1·m1(E1)·w2·m2·(E2)⋯·wn·mn(En).
  In this novel model, we choose SVM, BMPM, and BP as sensors, so the parameter *i* is define from 1 to 3. By formula (14), we can obtain the support degree of this network connection, *m*_123_(*N*), *m*_123_(*A*) and *m*_123_({*N*, *A*}) through fusion 3 sensors. This process needs that we should bring support degree for *N*, *A* and {*N*, *A*} computed by SVM, BMPM, and BP sensors, and weight vector* F-Score-N* and* F-Score-A *into formula (14).
(5) The final decision result by system is depicted in
  (15) $$\begin{array}{c} \text{Decision}\left(x\right)=\{\begin{array}{cc} \text{normal} & (\text{if}\;\;m_{123}\left(N\right)\\ & \;=\max\{m_{123}\left(N\right),m_{123}\left(A\right),\\ & \;\;\;\;\;m_{123}\left(\left\{N,A\right\}\right)\})\\ \text{abnormal} & (\text{if}\;\;m_{123}\left(A\right)\\ & \;=\max\{m_{123}\left(N\right),m_{123}\left(A\right),\\ & \;\;\;\;\;m_{123}\left(\left\{N,A\right\}\right)\})\\ \text{uncertain} & (\text{if}\;\;m_{123}\left(\left\{N,A\right\}\right)\\ & \;=\max\{m_{123}\left(N\right),m_{123}\left(A\right),\\ & \;\;\;\;\;m_{123}\left(\left\{N,A\right\}\right)\}). \end{array} \end{array}$$
  The final decision result can be explained in detail: if *m*_123_(*N*) is larger than *m*_123_(*A*) and *m*_123_({*N*, *A*}), this system considers current network connection as a normal one; as the same, if *m*_123_(*A*) is larger than *m*_123_(*N*) and *m*_123_({*N*, *A*}), this system considers current network connection as an abnormal one; if *m*_123_({*N*, *A*}) is larger than *m*_123_(*A*) and *m*_123_(*N*), this system cannot judge current network connection as a normal or abnormal one.

## 5. Experiments and Analysis

In this section, we would verify the effectiveness of combining ODS evidence theory with SVM, BMPM, and BP sensors and prove that this novel ODS network anomaly detection model can get higher detection rate (DR) and lower false positive rate (FR) for not only traditional attacks but also new attacks.

### 5.1. Data Set

#### 5.1.1. KDD99 Data Set

The KDD-Cup99 data set from UCI repository has been widely used as the benchmark data for network anomaly detection evaluation. It consists of several components depicted in Table 1. As in the case of the International Knowledge Discovery and Data Mining Tools Competition, only the “10% KDD” data is employed for the purposes of training. This contains 22 attack types and is essentially a more concise version of the “Whole KDD” data set. So in our experiments, we apply its 10% training data consisting of 494 021 connection records for training. Each connection record represents a sequence of packet transmission starting and ending at a time period and can be classified as normal traffic, or one of 22 different classes of attacks. All attacks fall into four main categories.

- Denial-of-service (Dos)—denial of the service that are accessed by legitimate users, for example, SYN flooding.
- Remote-to-local (R2L)—unauthorized access from a remote machine, for example, password guessing.
- User-to-root (U2R)—unauthorized access to gain local super-user (root) privileges, for example, buffer overflow attack.
- Probing (Probe)—surveillance and probing for information gathering, for example, port scanning.

The test data set has not the same probability distribution as the training data set. There are 4 new U2R attack types in the test data set that are not presented in the training data set. These new attacks correspond to 92.90% (189/228) of the U2R class in the test data set. On the other hand, there are 7 new R2L attack types corresponding to 63% (10196/16189) of the R2L class in the data set. In addition there are only 104 (out of 1126) connection records presented in the training data set corresponding to the known R2L attacks presented simultaneously in the two data sets. However there are 4 new Dos attack types in the test data set corresponding to 2.85% (6555/229853) of the Dos class in the test data set and 2 new Probing attacks corresponding to 42.94% (1789/4166) of the Probing class in the test data set.

#### 5.1.2. Data Set Preprocessing

Since a connection record in KDD 99 includes not only symbol feature but also continuous and discrete features, we must cope with these features before do experiments. Here Naïve algorithm in Rosetta software [17] is used to deal with continuous feature, and symbol feature can be discretized by general mapping method directly. Then in order to remove different features of various data and achieve general feature and same weights for discretized data, these data should be standardized, and these standardization formulas are introduced as follows:

(16) $$\begin{array}{c} {\text{I}}_{j}^{e}=\frac{1}{n}\overset{n}{\underset{i=1}{\sum }}{\text{I}}_{ij},\\ {\text{I}}_{j}^{\delta }=\sqrt{\frac{1}{n}\overset{n}{\underset{i=1}{\sum }}{\left({\text{I}}_{ij}-{\text{I}}_{j}^{e}\right)}^{2}},\\ {\text{I}}_{j}^{\prime }=\frac{{\text{I}}_{j}-{\text{I}}_{j}^{e}}{{\text{I}}_{j}^{\delta }}. \end{array}$$

### 5.2. Experimental Design

In order to prove that network anomaly detection system with ODS and RBPA has better performance, we design 4 kinds of experiments.

The first experiment is that we choose 3 single methods (SVM, BMPM, and BP) and 4 fusion methods (DS with SBPA, DS with RBPA, ODS with SBPA, and ODS with RBPA) to do detection in the same data set. In this data set, 4000 network connections of each connection type (Normal, Dos, Probe, and R2L) are selected and 249 network connections are chose from U2R type. These data chosen constructed a data set which is divided into 2 parts: training data set and test data set. This experiment is used to prove that the method we presented can detect various attacks and has higher DR and lower FR.

The second experiment is that we also choose these 7 network anomaly detection methods to do detection in R2L data set which has 4000 network connections. And the former 2000 network connections are normal connections and the later 2000 network connections are abnormal connections. This experiment is utilized to prove that the method with RBPA outperforms the method with ODS, and two optimization methods we presented can be used in network anomaly detection simultaneously with better performance.

The third experiment is that we also choose these 7 network anomaly detection methods to do detection in the same data set, like the first experiment. But we compare several parameters mentioned in Section 4.3, such as Precious, Recall, and* F-Score*. In addition, we utilize ROC curve which shows DR and FR of corresponding method, and AUC which represents the area under corresponding ROC curve to estimate the performance of network anomaly detection system.

The fourth experiment is that we choose 2 network anomaly detection methods (ODS with RBPA and DS with SBPA) to do detection. But here we choose 10% KDD99 data as training data set and test data set mentioned in Section 5.1.1 as test data set. This experiment is used to estimate the new model's detection ability for new attack type.

#### 5.2.1. Experiments with 4 Attack Types

From Tables 2, 3, 4, 5, 6, and 7, we can see that the false positive rate (FR) of single detection model, such as SVM, BMPM, and BP, is higher than that of fusion detection model. This reflects that fusion detection method can effectively reduce the FR in the anomaly detection system. From detection rate (DR) and attack number detected by anomaly detection methods, the DR of fusion detection method outperforms that of single method, and fusion method will bring lower FR. In addition, compared to fusion detection model, the variance of almost single detection model is larger, meaning that fusion detection model is not easy to shake, that is, relatively stable. Though the DR of some models for various attacks is high, its FR is still high, for example, BMPM model. Therefore, this novel model with ODS and regression BPA outperforms than others, and it has lower FR and better DR.

Here, we not only analyze the whole performance of this novel model, but also discuss ODS with weights and regression BPA performance. According to whether BPA and DS evidence theory redesigned, we can achieve 4 results for different combinations shown in Tables 5, 6, 7, and 8 respectively, including DS with Simple BPA (SBPA), ODS with SBPA, DS with regression BPA (RBPA) and ODS with RBPA.

According to whether DS evidence theory redesigned (whether adding weights into DS), we can divide these models into two groups without considering BPA design: one is Tables 5 and 6, and another is Tables 7 and 8. In this way, we can compare the performance of ODS, DS with weights (*F-Score* value as weights) with that of DS. From these two groups, we can see that the FR of ODS is lower than that of DS, and the total DR of ODS is also lower than that of DS. Clearly, most of DR of various attack types with ODS outperform that with DS. Thus, ODS with weights is effective compared with DS.

Similarly, according to whether BPA redesigned (whether with sensors' regression ability), we can divide these models into two groups without considering DS design: one is Tables 5 and 7, and another is Tables 6 and 8. In this way, we can compare the performance of RBPA with that of SBPA. For FR and total DR, RBPA is better than SBPA significantly. So RBPA with sensors' regression ability is effective compared with SBPA.

#### 5.2.2. Experiments with R2L Attack

**Figure 2:**
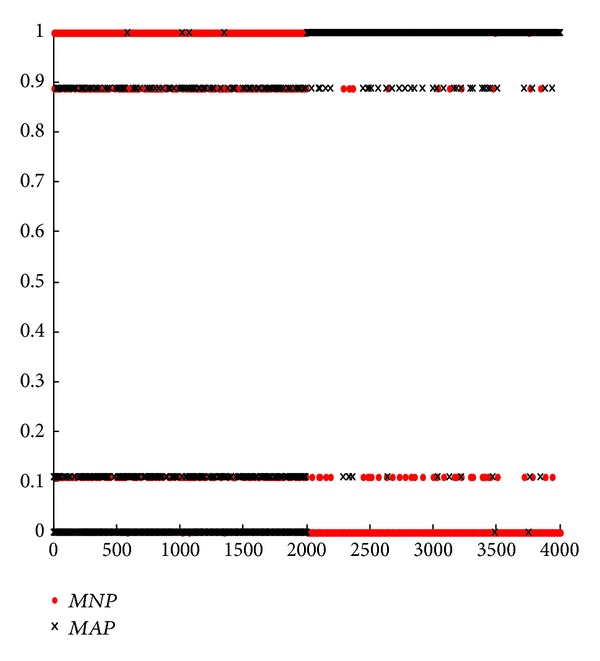
Results of classic DS evidence theory fusion of SBPA.

**Figure 3:**
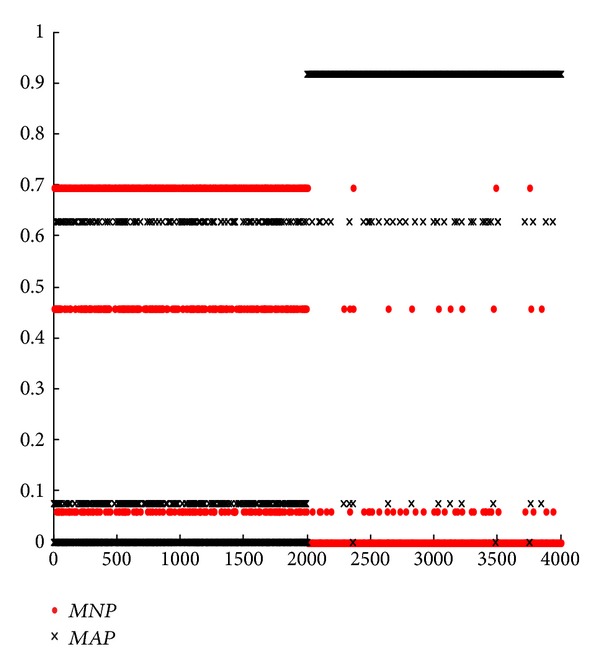
Results of ODS evidence theory fusion of SBPA.

**Figure 4:**
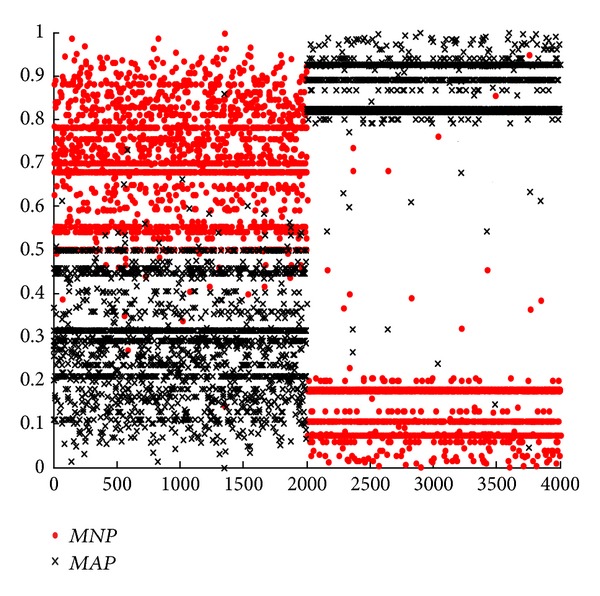
Results of classic DS evidence theory fusion of RBPA.

**Figure 5:**
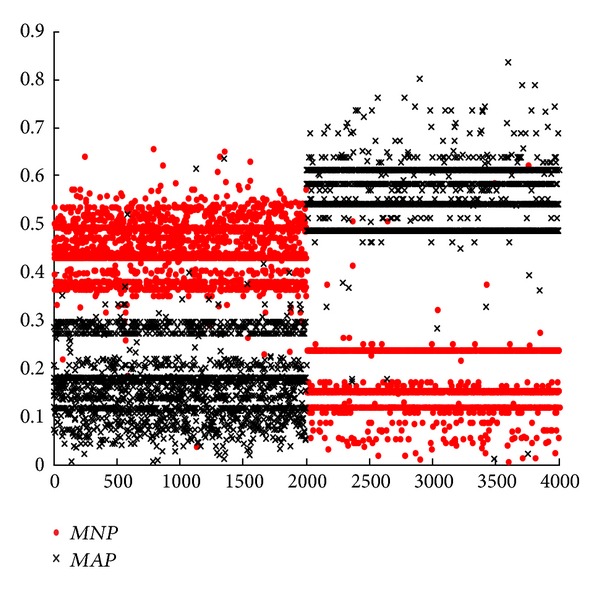
Results of ODS evidence theory fusion of RBPA.

In this subsection, we mainly focus on the novel model for single attack type according to formula (15). From Figures 2, 3, 4, and 5, we can see that they are achieved by different groups with redesigned or conventional BPA and DS. In these figures, corresponding with *m*_123_(*N*) in formula (15), parameter* MNP* represents the support degree of normal connection for current network connection after it is detected by SVM, BMPM, and BP sensors and merged by ODS. On the contrary,* MAP* corresponds with *m*_123_(*A*). By formula (15), if* MNP* is larger than* MAP*, current network connection is considered as a normal one, and vice versa. In this experiment, there are 4000 network connections in each figure, and the former 2000 network connections are normal connections and others are abnormal connections.

First, we analyze and compare Figures 4 and 5 in one group. In Figure 4, some normal connections of the former 2000 network connections overlap together for* MNP* and* MAP*. Significantly, some parts of* MAP* are above* MNP*, that is, this normal network connection is wrongly considered as an abnormal one, leading to a higher FR. On the contrary, the overlap in Figure 5 is less than that in Figure 4. In this way, Figure 3 outperforms Figure 2 with* MNP* and* MAP*. Without considering BPA design, ODS with weights is further effective.

Next, we analyze and compare Figures 2 and 4 in one group. In Figure 2, almost all the normal connections (the former 2000) overlap together for* MNP* and* MAP*. However, this overlap is further less in Figure 4. Clearly, this also occurs in the later 2000 connections, abnormal connections. In the same way, Figure 5 is better than Figure 3. In essence, this shows that RBPA method outperforms SBPA method, with lower FR and higher DR. This conclusion is consistent with the results from Tables 5 and 7 or Tables 5 and 8. Without considering DS design, RBPA with regression is further effective compared with SBPA.

Moreover, based on Figure 2, we compare Figure 3 with Figure 4. We can see that the results of* MNP* and* MAP* are distinguished easily and are suitable for real network better in Figure 4. But the opposite results are obtained in Figure 3, meaning that fuzzy and unseparated results. This leads a higher FR. Only verifying a condition, DS or BPA design, we can get Figure 3 with ODS optimization and Figure 4 with RBPA optimization. From Figures 3 and 4, we conclude that RBPA method is better than ODS method in network anomaly detection system.

Similarly, based on Figure 2, we compare Figures 3 and 4 with Figure 5. We can see that the result of Figure 5 is better than both Figures 3 and 4 significantly. Compared with Figure 2, Figure 5 is improved enormously. In a word, no matter which one system chooses, the performance of optimized network anomaly detection system will be improved clearly. Specially, these two optimization methods can be utilized by network anomaly detection system simultaneously, leading a better result than the one with either optimization method.

#### 5.2.3. Experiments Based on ROC and AUC

**Figure 6:**
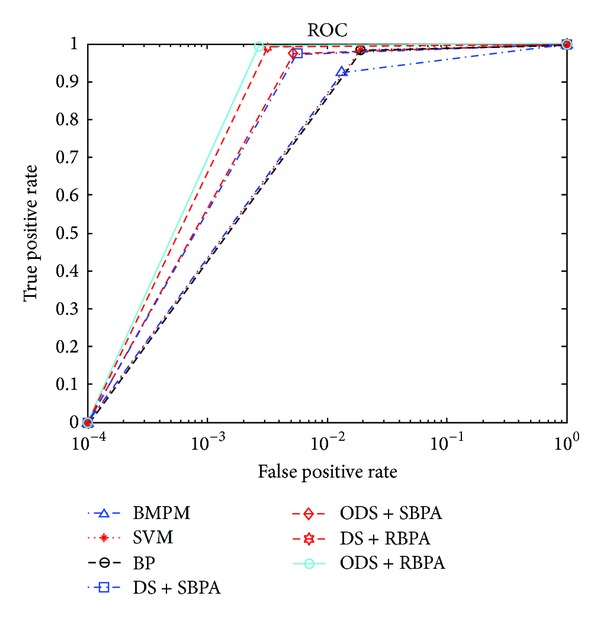
The ROC curve of BMPM, SVM, BP, DS with SBPA, ODS with SBPA, DS with RBPAS, and ODS with RBPA.

Table 9 shows that the results of all network normal and abnormal connections used by various anomaly detection methods. And the ROC curve of each method is depicted in Figure 6. In these two experiments, we employ ROC curve that shows the relationship of FR and DR, and AUC that represents the area under ROC curve. Here several parameters are utilized to estimate network anomaly detection system, such as Precious, Recall, and* F-Score* which are introduced in Section 4.3. Specially, the larger the values of parameters (*F-Score*, AUC) are, the better the performance of corresponding system is.

First, compared single detection methods, SVM, BMPM, and BP with fusion detection methods, we can see that single detection methods have smaller values of* F-Score* and AUC from Table 9; that is, the performance of single detection methods is lower than that of fusion methods. Ensuring an invariable condition in 4 fusion methods, we can analyze the effectiveness of RBPA and ODS. In this way, compared DS with SBPA and DS with RBPA, ODS with SBPA and ODS with RBPA, we can see that the methods with RBPA have higher* F-Score* and AUC values. As the same, the methods with ODS have higher* F-Score* and AUC values.

From Figure 6 that shows the ROC curve of 7 network anomaly detection methods, the network anomaly detection method merged with ODS and RBPA has the largest area under corresponding ROC curve (the largest AUC value in Table 9). When they have the same DR, FR of the network anomaly detection method merged with ODS and RBPA is the smallest one. Similarly, when they have the same FR, DR of the network anomaly detection method merged with ODS and RBPA is the highest one. So this fusion method is the best one in these 7 fusion methods.

#### 5.2.4. Experiments with New Attacks

In this experiment, we utilize 3 network anomaly detection systems (BP, DS with SBPA and ODS with RBPA) to detect new attacks. Unlike experiments mentioned above, the data set used in this experiment is 10% KDD99 and test data set with 17 new attack types in Section 5.1.1.

From Table 10, we can see that the performance of single method is lower than that of fusion method. Most of new attack connections DR are higher than BP, but there still exist some abnormal DR, “sqlattack” for example. With ODS and RBPA optimization, this novel method we presented makes up this defect, which has a better new attack detection performance than others.

## 6. Related Work

The use of data fusion in the field of network anomaly detection is presented by Siaterlis and Maglaris [18]. The Dempster-Shafer theory of evidence is used as the mathematical foundation for the development of a novel anomaly detection engine. The detection engine is evaluated using the real network traffic. The superiority of data fusion technology applied to intrusion detection systems is presented in the work of Wang et al. [19]. This method used information collected from the network and host agents and application of Dempster-Shafer theory of evidence. Another work incorporating the Dempster-Shafer theory of evidence is by Hu et al. [20]. Wu et al. [21], proposed a framework of client-server architecture where the mobile agent continuously extracted various features and send to the server to detect anomaly using anomaly detectors. They used multiple distributed servers with different machine learning as a detector for analyzing the feature vector and D-S Evidence theory of information fusion is used to fuse the results of detectors, also proposed a cycle-based statistical approach to find anomaly activity. Zhouzhou et al. [22] presented a new algorithm based on D-S evidence theory to reduce energy consumption in wireless sensors network, which modifies D-S evidence theory and fuses it on cluster-head selection phase and adjusts operation period. The Dempster-Shafer theory of evidence in data fusion is observed to solve the problem of how to analyze the uncertainty in a quantitative way.

Reference [11] presented a novel intrusion detection approach combining SVM and KPCA to enhance the detection precision for low-frequent attacks and detection stability. In order to shorten the training time and improve the performance of SVM classification model, an improved radial basis kernel function (N-RBF) based on Gaussian kernel function is developed, and GA is used to optimize the parameters of SVM. [14] proposed a flow-based anomaly detection system, which is trained with a flow-based data set. In this new system, multilayer Perceptron neural network with one hidden layer is used, which is added interconnection weights by a Gravitational Search Algorithm. Giacinto et al. [23] utilized general classifiers to divide various feature subspaces from the same data set and then merged voting, mean algorithm, Bayes, and decision module together. However, there exists less analysis about detection algorithm and fusion method. Another drawback of this model is higher false alarm rate. The formulation of the intrusion detection problem as a pattern recognition task using data fusion approach based on multiple classifiers is attempted by Didaci et al. [24]. The work confirms that the combination reduces the overall error rate, but may also reduce the generalization capabilities. Ambareen Siraj et al. [25] brought fuzzy cognitive map into fusion network anomaly detection and presented an intelligent network anomaly detection model. Thomas and Balakrishnan [26–28] selected artificial neural network as fusion algorithm and constructed fusion network anomaly detection model based on SNORT, PHAD, and ALAD that are open source detection systems. Although it was proved as an effective system, but its detection rate for some attacks was lower. In [29], performance of this fusion model is decided by diversity of various classifiers. [30] presented a novel network anomaly detection system with DS evidence theory and regression neural network, but its detection rate is lower.

## 7. Conclusion and Future Work

In this paper, we present a novel network anomaly detection model with ODS evidence theory and RBPA. When applying DS evidence theory on network anomaly detection model, we set weight for each sensor. And the weight value is from prior knowledge,* F-Score*-*N* and* F-Score-N,* which are extended by* F-Score*. Another key contribution is a new BPA function. We utilize regression ability of classifiers, SVM, BMPM, and BP, to compute various support degrees (*m*(*N*), *m*(*A*) and *m*({*N*, *A*})) for each status (normal, abnormal, and unknown). Finally, we design 4 kinds of experiments to prove that this novel network anomaly detection model has higher DR and lower FR.

To further improve the performance of this new model, we will choose other sensors as classifiers to cope with complicated network records. And we will also utilize new attacks to evaluate this model. Finally, we will try to optimize DS evidence theory according to features of sensors.

## Figures and Tables

**Table 1:** KDD data set.

Data set	Total	Normal (%)	DOS (%)	Probe (%)	U2R (%)	R2L (%)
10% KDD	494,020	19.79	79.2	0.8	0.01	0.2
Test KDD	311,029	19.58	73.9	1.3	0.02	5.2
Whole KDD	4,898,430	19.8	79.3	0.84	0.001	0.02

**Table 2:** The detection result of all the attacking types with SVM (FR = 1.91%).

Attack type	Attack number	Attack number detected	DR (%)
DOS	2000	1989	99.45
Probe	2000	1992	99.6
U2R	124	102	80.64
R2L	2000	1950	82.20

Total	6124	6033	98.51
Variance			9.76

**Table 3:** The detection result of all the attacking types with BP (FR = 1.90%).

Attack type	Attack number	Attack number detected	DR (%)
DOS	2000	1971	98.55
Probe	2000	1986	99.30
U2R	124	95	76.61
R2L	2000	1974	98.70

Total	6124	6026	98.39
Variance			9.90

**Table 4:** The detection result of all the attacking types with BMPM (FR = 1.32%).

Attack type	Attack number	Attack number detected	DR (%)
DOS	2000	1931	96.55
Probe	2000	1982	99.10
U2R	124	115	92.74
R2L	2000	1987	99.35

Total	6124	6015	98.22
Variance			2.72

**Table 5:** The detection result of all the attacking types with classic DS evidence theory fusion of simple BPA method (FR = 0.62%).

Attack type	Attack number	Attack number detected	DR (%)
DOS	2000	1987	99.35
Probe	2000	1990	99.50
U2R	124	116	93.54
R2L	2000	1986	99.30

Total	6124	6079	99.26
Variance			2.60

**Table 6:** The detection result of all the attacking types with ODS evidence theory fusion of Simple BPA method (FR = 0.59%).

Attack type	Attack number	Attack number detected	DR (%)
DOS	2000	1985	99.25
Probe	2000	1993	99.65
U2R	124	118	95.16
R2L	2000	1987	99.35

Total	6124	6083	99.33
Variance			1.90

**Table 7:** The detection result of all the attacking types with classic DS evidence theory fusion of regression BPA method (FR = 0.32%).

Attack type	Attack number	Attack number detected	DR (%)
DOS	2000	1997	99.85
Probe	2000	1991	99.55
U2R	124	116	93.54
R2L	2000	1994	99.70

Total	6124	6098	99.57
Variance			2.74

**Table 8:** The detection result of all the attacking types with ODS evidence theory fusion of regression BPA method (false positive rate = 0.27%).

Attack type	Attack number	Attack number detected	DR (%)
DOS	2000	1997	99.85
Probe	2000	1997	99.85
U2R	124	119	95.97
R2L	2000	1992	99.60

Total	6124	6105	99.69
Variance			1.69

**Table 9:** Comparison of BMPM, SVM, BP, DS, ODS, SBPA, and RBPA.

Detection method	Precision	Recall	*F-Score*	AUC

BMPM	0.9835	0.9263	0.9540	0.9851
SVM	0.9752	0.9851	0.9801	0.9780
BP	0.9753	0.9839	0.9796	0.9781
DS with SBPA	0.9926	0.9748	0.9836	0.9934
ODS with SBPA	0.9933	0.9749	0.9840	0.9940
DS with RBPA	0.9957	0.9910	0.9934	0.9962
ODS with RBPA	0.9964	0.9930	0.9947	0.9968

**Table 10:** Comparison of BP, DS with SBPA and ODS with RBPA for new attacks.

Attack name	Total connections	Detected connections			DR (%)		
		BP	DS + SBPA	ODS + RBPA	BP	DS + SBPA	ODS + RBPA
Apache2	794	792	794	794	99.75	100.00	100.00
httptunnel	158	155	157	158	98.10	99.37	100.00
mailbomb	5000	4893	5000	500	97.86	100.00	100.00
mscan	1053	1050	1050	1052	99.72	99.72	99.91
named	17	16	17	17	94.12	100.00	100.00
processtable	759	758	759	759	99.87	100.00	100.00
ps	16	16	16	16	100.00	100.00	100.00
saint	736	736	735	736	100.00	99.86	100.00
sendmail	17	14	16	17	82.35	94.12	100.00
snmpgetattack	7741	7704	7716	7739	99.52	99.68	99.97
snmpguess	2406	2404	2404	2406	99.92	99.92	100.00
sqlattack	2	2	1	2	100.00	50.00	100.00
udpstorm	2	0	2	2	0.00	100.00	100.00
worm	2	2	2	2	100.00	100.00	100.00
xlock	9	7	6	8	77.78	66.67	88.89
xsnoop	4	3	4	4	75.00	100.00	100.00
xterm	13	13	13	13	100.00	100.00	100.00

Average					89.65	94.67	99.34
